# Rationale and design for Healthy Hearts in Manufacturing (HHM): A pragmatic single-arm hybrid effectiveness-implementation study for hypertension management and tobacco cessation

**DOI:** 10.1016/j.conctc.2025.101444

**Published:** 2025-02-03

**Authors:** Hanzi Jiang, Yao Tian, Jennifer Bannon, Amy E. Krefman, Lawrence C. An, Dustin D. French, Claude R. Maechling, Jane Holl, Richard Chagnon, Theresa L. Walunas, Christopher Burch, Anthony Musci, Darce Latsis, Dawn Carey, Megan McHugh

**Affiliations:** a Institute for Public Health and Medicine, Northwestern University, Feinberg School of Medicine, Chicago, IL, USA; b Department of Surgery, Feinberg School of Medicine, Northwestern University, Chicago, IL, USA; c Department of Preventive Medicine, Feinberg School of Medicine, Northwestern University, Chicago, IL, USA; d Department of Internal Medicine, University of Michigan, Ann Arbor, MI, USA; e Departments of Ophthalmology and Medical Social Sciences, Feinberg School of Medicine, Chicago, IL, USA; f Department of Veterans Affairs Health System Research Service, Hines, IL, USA; g Branstad Family Foundation, Chicago, IL, USA; h Department of Neurology, Biological Sciences Division and Center for Healthcare Delivery Science and Innovation, University of Chicago, Chicago, IL, USA; i OnSite Care, a CareATC Affiliate, Salt Lake City, UT, USA; j Perdue Farms, Salisbury, MD, USA

**Keywords:** Quality improvement, Tobacco cessation, Hypertension, Worksite health centers

## Abstract

**Background:**

Heart disease is the leading cause of mortality in the United States and contributes more than $320 billion annually in health care costs and lost productivity. Manufacturing employment is associated with higher rates of hypertension and smoking. Many large manufacturers provide health services to employees and their family members through worksite health centers (WHCs). Several quality improvement interventions for hypertension and tobacco cessation have shown to be effective in community-based primary care sites. The Healthy Hearts in Manufacturing (HHM) study aims to implement and test these interventions in WHCs.

**Methods:**

Two organizations that operate WHCs at manufacturing sites volunteered to participate in the 58-month HHM research study. The HHM intervention involves monthly coaching to assist WHCs with implementing evidence-based strategies for hypertension management and tobacco cessation advocated by the Million Hearts initiative and the U.S. Preventive Services Task Force. A pragmatic, Type II hybrid effectiveness-implementation study design is used to evaluate HHM. The approach is inspired by the stepped-wedge cluster-randomized trial to assess intervention effectiveness. We will conduct interviews to identify facilitators and barriers to implementation and budget impact analysis to estimate the financial impact of the HMM interventions and the potential healthcare savings to companies and Medicare.

**Results:**

Twelve WHCs were randomly selected to enroll in HHM. The WHCs are in nine states and provide primary care services for employees and family members of four manufacturing companies. Baseline patient smoking rates ranged from 13 % to 59 % across WHCs. The percentage of patients with blood pressure of 140/90 or greater ranged from 7 % to 56 % across WHCs.

**Conclusion:**

This exploratory five-year research study will identify facilitators and barriers to implementing the HHM interventions in WHCs, evaluate the effectiveness of hypertension management and use of tobacco screening and cessation, and provide evidence of HHM's potential cost-effectiveness for employers and Medicare.

## Highlights

• Manufacturing workers have high rates of heart disease, and worksite health centers (WHC) are well-positioned to intervene.
• Healthy Hearts in Manufacturing aims to improve tobacco cessation and hypertension care in 12 randomly selected WHCs.
• The evaluation is inspired by a stepped-wedge, cluster-randomized trial design.
• The research study will also provide estimates of the intervention's healthcare cost savings for employers and Medicare.

## 1 Background

Heart disease is the leading cause of death in the United States (US) and contributes more than $320 billion annually in healthcare expenses and lost productivity [1,2]. Manufacturing communities, which are largely small and rural, have a significantly higher incidence of heart disease and its risk factors, such as smoking and obesity, compared to non-manufacturing communities [[3], [4], [5]]. Currently, there are 13 million manufacturing workers in the US; they are predominantly male and older than workers in other industries [6,7]. Jobs in manufacturing are also associated with higher rates of hypertension, physical inactivity, inadequate sleep, binge drinking, and fewer quality-adjusted life years [[8], [9], [10], [11], [12]].

Many large manufacturing companies, particularly those located in areas with limited primary care availability, provide health services for employees, spouses, dependents, and retirees through worksite health centers (WHCs) [13,14]. According to a recent national survey, 31 % of employers with over 5000 employees offer WHCs [15]. The WHCs usually have a limited staff that typically includes a nurse practitioner (68 %), physician (66 %), medical assistant (62 %), and/or nurse (53 %) [15]. Many WHCs are designed to function as primary care medical homes, emphasizing accessible care [15]. To encourage utilization of WHC services, many do not charge patients for care or charge only a nominal fee. Given their affordability and accessibility, WHCs are well-positioned to address chronic disease, poor health behaviors (e.g., smoking), and other health deficiencies prevalent in the US manufacturing communities.

Healthy Hearts in Manufacturing (HHM) is a 58-month (September 2023–December 2028) research study that seeks to provide coaching to 12 WHCs based at manufacturing sites to implement evidence-based quality improvement (QI) interventions related to hypertension management and tobacco cessation. HHM is testing the use of a coaching model as the strategy to support WHCs to implement best practices and improve their capacity for QI. The coaching includes health information technology support; data, feedback, and benchmarking; and expert consultation. These supports are delivered through practice facilitation, meaning a coach works with each participating WHC to help the WHCs make meaningful changes. HHM's approach to QI expands and builds upon successful elements of Healthy Hearts in the Heartland [16], Healthy Hearts in Michigan [17], and the Agency for Healthcare Research and Quality's EvidenceNOW initiative [18], which introduced coaching and the QI interventions in community-based primary care. HHM is an exploratory study to implement and test the coaching and QI interventions in WHCs for the first time. It is supported by a Large Research Demonstration and Dissemination (R18) grant from the Agency for Healthcare Research and Quality, which is designed to foster the application of existing knowledge.

HHM includes three components: (1) adapting the evidence-based hypertension management and tobacco cessation recommendations for use in WHCs, (2) evaluating the ability of WHCs to implement components of the HHM intervention by identifying facilitators and barriers to implementation and testing the effectiveness of the model to improve hypertension management and tobacco use screening and cessation, and (3) estimating the financial impact of HHM and its potential for health care savings to manufacturing companies and Medicare. This paper focuses on the second and third components. Here, we describe the inclusion, exclusion, and randomization of WHCs; the approach to identifying facilitators and barriers to implementation; and the analysis plan to examine our hypotheses and estimate the budget impact of HHM.

## 2 Material and methods

### 2.1 Study setting and participants

WHCs were recruited in advance of the study with assistance from the National Association of Worksite Health Centers, which hosted a webinar for members to learn about the study. WHCs were eligible if they provide primary care (i.e., not just occupational health or vaccines) and use an electronic health record (EHR) capable of extracting data needed for reporting outcome measures used in this study. Two organizations that operate WHCs volunteered to participate in HHM, and they are identified as Companies A and B in this paper to facilitate confidentiality. Company A is among the 50 largest private companies in the U.S. and operates WHCs for employees and dependents at its manufacturing sites. Company B is a health care organization that operates WHCs for multiple companies, including several manufacturers.

### 2.2 HHM interventions

HHM interventions are evidence-based and focus on the clinical areas of hypertension management, tobacco screening and cessation, advocated by the Million Hearts initiative and the U.S. Preventive Services Task Force [19]. Specifically, HHM includes clinical interventions in four areas: improving accurate blood pressure measurement and treatment, identifying patients with hypertension and smokers through electronic health record (EHR) registries, self-monitoring of blood pressure (SMBP) programs, and an “Ask-Advise-Connect” approach to support tobacco cessation. Here, tobacco is defined as any tobacco-derived product for human consumption, including cigarettes, cigars, smokeless tobacco, and electronic nicotine delivery systems like vapes and e-cigarettes [20]. A list of QI strategies associated with the interventions is shown in Table 1.

**Table 1:** Healthy hearts in manufacturing interventions, quality improvement (QI) strategies, and facilitation activity examples.

HHM Intervention	QI Strategies	Examples of Facilitation Activities
Improving accurate blood pressure (BP) measurement and treatment	• Training on accurate BP measurement (AMA guidelines) • Implement BP treatment protocol • Implement alerts or clinical decision supports for patients with uncontrolled BP • Encourage use of validated, automated BP cuffs for measurement	• Measurement of patient BP at least stressful times • Strategic placement of instructions for accurate BP measurement • Assessment of potential delivery of patient education by portal/SMS message • Training on BP on pharmacotherapy • Training on patient shared decision making/motivational interviewing • Redesign WHC workflow to facilitate accurate BP measurement • Assessment of WHC barriers to BP protocol adoption • Engagement of WHC leadership to obtain validated, automated BP cuffs • Identification and dissemination of WHC prescribed BP management (i.e., coverage, available on site, distribution during the workday)
Identify patients with hypertension and smokers through electronic health record (EHR) registries	• Generate a list of patients with uncontrolled BP • Generate a list of patients who smoke • Implement a protocol for outreach and follow up for patients with hypertension or who smoke	• Assessment of EHR documentation in structured data fields for tobacco use screening and BP measurement • Work with information technology/leadership/health center on data field documentation to capture data required for reports • Promote use of decision tools during visit and/or electronically • Assessment of workflows to facilitate follow up visits and document in EHR
Self-monitoring of blood pressure (SMBP) programs	• Design and implement workflows for patients to perform and report home BP measurements • Design and implement workflows for WHC clinicians to educate patients on SMBP	• Engagement of WHC leadership establish SMBP program (e.g., BP cuff loan program, funds for new home monitors)
An “Ask-Advise-Connect” approach to support tobacco cessation.	• Screen patients for tobacco use status • Document status in the EHR • Implement clinical alerts/decision support for patients who identify as smokers • Offer tobacco cessation interventions (i.e. counseling, pharmacotherapy, Quit line) and documenting in EHR • Document tobacco cessation interventions offered in the EHR • Redesign workflows to provide tobacco cessation counseling and/or prescribing • Implement referrals to state quit line; close the “referral loop”	• Assessment of “closure” of state quit line referral loop • Assessment of feasibility of electronic delivery of education (portal/SMS messages) • Training on pharmacotherapy for tobacco cessation • Training on patient shared decision tools/motivational interviewing • Training on counseling vs. advice for tobacco cessation • Identification of barriers to tobacco cessation protocol adoption • Identification and dissemination of WHC prescribed tobacco cessation medication options (i.e., coverage, available on site, distribution during the workday)
		**Facilitation Activities Related to All Four Clinical Interventions**
		• Assess availability/access to health center before/during/after shift • Assess wellness incentives/alignment with company goals • Work with leadership to promote incentive/raffle programs • Assess health center relationship with sponsoring company • Develop community resource list • Instruct on QI methodology

AMA = American Medical Association; BP = blood pressure; MAP = measure accurate blood pressure; EHR = electronic health record; QI = quality improvement.

The clinical interventions are reinforced through health information technology support, data, feedback, benchmarking, and expert consultation. These supports are delivered through practice facilitation, meaning each participating WHC receives personalized coaching to help the practices make meaningful changes. Practice facilitation is an evidence-based method to help primary care practices implement new approaches to improve the quality of care. It is associated with successful QI implementation and improvement in care delivery and chronic disease outcomes [[21], [22], [23], [24], [25], [26]]. One study found that practices are 2.8 times more likely to adopt evidence-based guidelines with practice facilitation [27]. Practice-facilitated strategies can improve care for those who regularly visit the clinic and also those who do not. For the HHM study, the practice facilitator is an experienced nurse with expertise in using health informatics to support QI. She has provided practice facilitation expertise on several EvidenceNOW projects that focus on similar clinical interventions for hypertension and tobacco cessation. Table 1 includes a list of activities that she will undertake associated with each HHM clinical intervention.

**Fig. 1:**
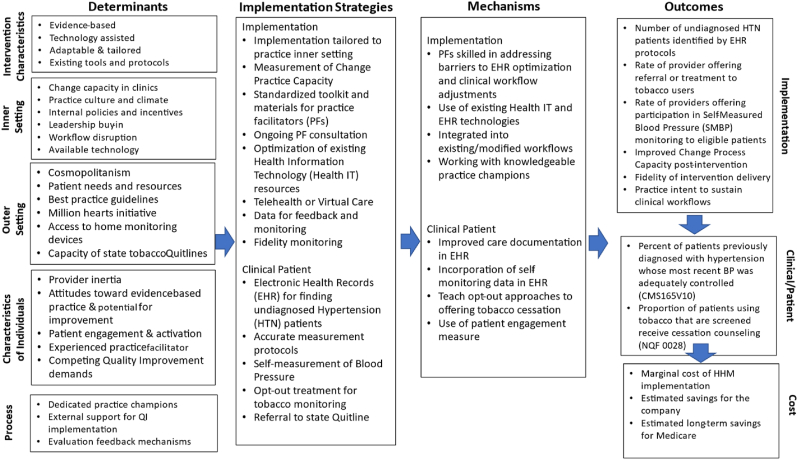
Implementation Research Logic Model for Healthy Hearts in Manufacturing (HHM) Description: Implementation Research Logic Model for HHM, outlining how specific determinants, such as intervention and individual characteristics, inform the HHM implementation strategy that drives the change mechanism, with the goal of improved outcomes.

Fig. 1 shows the Implementation Research Logic Model for HHM [28]. The Logic Model outlines how the specific determinants (intervention characteristics, inner and outer setting, process, and individual characteristics) inform the HHM implementation strategy that drives the change mechanisms, with the goal of leading to improved outcomes.

### 2.3 Study design

**Fig. 2:**
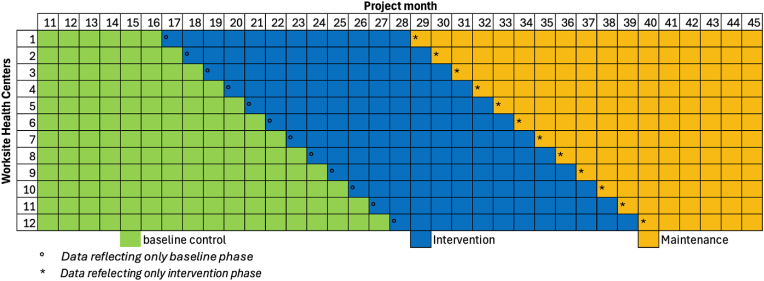
Healthy Hearts in Manufacturing (HHM) Study Timeline Description: The illustration of the study timeline for all participating worksite health centers inspired by the stepped-wedge cluster-randomized trial design.

The HHM study design is inspired by stepped-wedge, cluster-randomized trial but differs in key ways with its smaller sample size, exploratory nature, and pragmatic focus. It is best described as a pragmatic single-arm hybrid effectiveness-implementation study, emphasizing the implementation and testing of HHM in WHC's unique clinical setting and practical application [17]. Specifically, the intervention is administered and measured at the WHC level, and WHC-level aggregated data are retrieved from existing EHR registries. The first 10 months of the project are dedicated to logistic planning, interviews with WHC clinicians, and adaptation of HHM strategies. All WHCs begin in the baseline phase and then shift to the intervention phase at staggered time points. The baseline phase is the control condition, during which WHCs continue with their usual care for at least six months. The baseline phase begins in HHM's eleventh project month for all WHCs. The intervention phase, which lasts for 12 months, is when WHCs receive coaching on the HHM clinical interventions. Once the intervention phase is completed, each WHC will be moved to a maintenance phase, which lasts for at least six months. During such time, although WHCs will no longer receive any coaching, data collection will continue. Fig. 2 illustrates the study timeline for the participating WHCs. [NAME BLINDED FOR PEER REVIEW] Institutional Review Board (IRB) approved the HHM protocol and serves as the single IRB.

### 2.4 Randomization

We used stratified randomization based on WHCs' parent companies (i.e., Companies A and B) to ensure the balance of WHCs across treatment sequences. A total of 12 WHCs were selected, seven from Company A and five from Company B, determined by the companies’ capacity and willingness to participate. Within each company stratum, the timing of the implementation introduction was randomly allocated [29]. In an effort to minimize participation burdens on the Companies (i.e., company leaders participated in kick-off calls with the WHCs as they began the intervention), we alternated intervention start dates between WHCs from Companies A and B (starting with those with the smallest numbers) to balance rollout across the two companies over time. Company A was assigned to start first because it had more WHCs participating. All randomization was conducted in SAS (version 9.4, SAS Institute Inc, Cary, NC).

### 2.5 Data collection

Data will be collected from multiple sources, including EHRs; tracking documents recorded by the practice facilitator using the Facilitation Activity and Intervention Tracing System (FACITS); and through interviews with company leaders, clinicians, and staff at the WHCs. Throughout the study, no individual or patient-level data will be collected; only aggregated data at the WHC level will be included in the study.

WHC-level patient demographic information will be collected twice from all participating WHCs: three months before a WHC enters its intervention phase and one month after it completes its intervention period. The data will include the mean, median, first and third quantiles of the patient's body mass index (BMI) and systolic and diastolic blood pressure. We will also collect the number of unique patients in each age group per gender and the percentage of patients by race and ethnicity.

The practice facilitator will use FACITS to document the encounters with the WHCs and progress in implementing the QI strategies associated with the clinical interventions (Table 1). The FACITS platform includes forms to support the tracking of activities during each encounter, including visit duration, mode (in-person vs. virtual), type(s) of facilitation interventions engaged in, topics covered, participating clinic staff, and free text notes. Examples of some of the facilitation activities to be tracked in FACITS are shown in the third column of Table 1. Additionally, the practice facilitator will document progress on the implementation of each QI activity, noting whether each activity was not implemented and not intending to implement, not implemented but intending to implement, partially implemented, implemented but not sustained, and fully implemented and sustained. The practice facilitator will document the progress of each WHC quarterly in FACITS.

### 2.6 Identifying barriers and facilitators to sustainable maintenance of Healthy Hearts in manufacturing in worksite health clinics

Given the relatively small number of participating WHCs, we will pursue a qualitative approach to identify facilitators and barriers to sustainable implementation. Our data collection instruments will follow the logic model (Fig. 1) to explore the determinants and implementation strategies that facilitated or hindered sustainable implementation and, ultimately, changes in outcomes. We will assess barriers and facilitators at each of the twelve participating WHCs by conducting individual interviews with company leaders, WHC administrators and clinical staff. These interviews will be conducted toward the end of each WHC's intervention period. Interviews will be conducted via Zoom, professionally transcribed, and stored in Atlas.ti, a qualitative software program, for analysis [30]. To manage the data and assist in identifying patterns and themes, we will apply a combination of deductive codes based on the semi-structured interview protocol and inductive codes based on emergent concepts following an initial reading of the transcripts [31].

### 2.7 Evaluating the impact of HHM on hypertension management and tobacco screening and cessation

HHM uses two outcome measures derived from Clinical Quality Measures (CQM) from the Centers for Medicare and Medicaid Services (CMS): CMS165v10 (controlling high blood pressure) and CMS138v11 (tobacco screening and cessation intervention) (Table 2). These measures represent the HHM intervention's impact at the WHC level. The measures will be treated as proportions, with the numerator being the number of patients that met the measurement criterion and the denominator being the number of eligible patients during the measurement period. To calculate these two measures, several data components will be collected monthly from each participating WHC throughout the study period from the baseline through the maintenance phase, a total of 35 months. For each monthly data collection, we will use a 12-month look-back period, meaning that each data point represents aggregate outcomes from the previous 12 months. In the end, one data point related to hypertension management and one data point related to tobacco cessation will be collected per WHC per month, both reflecting data from the preceding 12 months. The 12-month look-back period was selected to align with the definition of the outcome measures and was determined to be feasible given the natural limitations of the participating WHCs' electronic health records systems. EvidenceNow projects using coaching and similar QI interventions have previously documented challenges associated with using EHR data to enable the calculation of performance measures [[32], [33], [34]]. Descriptive statistics will be calculated for all measurements monthly across the study period, including means and standard deviations, and ranges, or counts and percentages, as appropriate.

**Table 2:** Primary outcome measures and related data elements.

Measure	CMS165v10 [35]	CMS138v11 [20]
Measure Definition	Percent of patients, 18–85 years of age, who had a diagnosis of essential hypertension starting before and continuing into, or starting during the first 6 months of the measurement period, and whose most recent BP was adequately controlled (<140/90 mmHg) during the measurement period.	Percentage of patients aged 18 years and older who were screened for tobacco use one or more times during the measurement period AND who received tobacco cessation intervention during the measurement period if identified as a tobacco user.^a^
Data Elements Collected Monthly from Electronic Health Records	1.The number of adults (18–85) who: had at least one visit to the health center during the measurement period; AND had at least one blood pressure measure recorded during the measurement period	
	2. The number of adults in (1) who have ever been diagnosed with hypertension	4.The number of adults in (1) who were screened for tobacco use during the measurement period
	3. The number of adults in (2) whose most recent systolic blood pressure measurement is ≤ 139 AND diastolic blood pressure measurement is ≤ 89	5. Number of adults in (4) who were identified as a tobacco user
		6. Number of adults in (5) who received a tobacco cessation intervention (including counseling and/or pharmacotherapy) during the measurement period.
Denominator	(2) Adults with hypertension	(1) Initial Population – rate 1
Numerator	(3) Controlled hypertension	(4) Adults who screened for tobacco use – rate 1

a Three rates are reported for CMS138v11: (1) Percentage of patients aged 18 years and older who were screened for tobacco use one or more times during the measurement period, (2) Percentage of patients aged 18 years and older who were identified as a tobacco user during the measurement period who received tobacco cessation intervention during the measurement period or in the six months prior to the measurement period, and (3) Percentage of patients aged 18 years and older who were screened for tobacco use one or more times during the measurement period AND who received tobacco cessation intervention during the measurement period or in the six months prior to the measurement period if identified as a tobacco user.

#### 2.7.1 Statistical analysis plan

The primary outcomes of interest analyzed are WHC-level CMS165 V10 and CMS138v11. Due to the outcomes of this study representing aggregate outcomes from the previous 12 months, we will use data collected from the first month of intervention (reflecting only baseline phase data) to compare with data collected in the first month after completion of the intervention (reflecting the 12-month intervention). This approach minimizes contamination from overlapping periods and ensures a clear comparison between the baseline and the 12-month intervention. In the main analysis, we will utilize a paired *t*-test to evaluate whether there is a significant improvement in the study outcomes between the baseline and the 12-month intervention [36]. Each WHC serves as its own control in the paired analysis, and the differences in study outcomes between the baseline and the 12-month intervention are assumed to follow normal distributions [37]. The paired *t*-test will test the null hypothesis that there are no significant differences in outcomes between two time points. A p-value of <0.05 will indicate a significant change in outcomes, implying the effectiveness of the intervention. If the normality assumption is violated, we will consider a non-parametric alternative, such as the Wilcoxon signed-rank test [38]. The paired *t*-test will test the null hypothesis that there are no significant differences in outcomes between two time points. A p-value of <0.05 will indicate a significant change in outcomes, referring to the effectiveness of the intervention.

We will conduct two sensitivity analyses to account for potentially underlying temporal trend in the study outcomes and to explore “time-on-treatment effect” by incorporating more data points collected before and after intervention and leveraging the time-on-treatment effect model proposed by Hughes et al. [39].

$$\mu_{ij}=\mu +\mu_{i}+\beta_{j}+\theta \left(s_{ij}\right)\;X_{ij}$$

where $\mu_{ij}$ represents the expectation of the outcome, $y_{ij}$, (e.g., CMS165v10) at WHC *i* at time point *j*; μ is an intercept term that denotes the grand mean of the outcome at baseline; the random intercepts, $\mu_{i}$, represents each cluster's unique baseline; the effect of time is modeled as a categorical variable, and its fixed effect is represented by $\beta_{j}$; $X_{ij}$ is the indicator of intervention phase for WHC i at time point j; $\theta \left(s_{ij}\right)$ is the intervention effect as a function of the exposure time in the intervention phase, with $s_{ij}$ indicating the exposure time of intervention for cluster i at time j. The model is further illustrated in the Supplementary Figure.

Given the nature of the data collection procedure in this study, we assume that the intervention effect would be gradually reflected by the outcome data collected over time (i.e., a linear time-on-treatment effect reflected by $\theta \left(s_{ij}\right)=\left(\theta *s\right)$ in the Sensitivity Analysis 1. In this scenario, *s* denotes the exposure time of the intervention for cluster i at time j, and the maximum number of periods that a cluster can be exposed under intervention is 12 months; θ is the monthly fractional effect for a WHC [39]. Further, we will add an indicator for the parent companies of WHCs (0 = Company A; 1 = Company B) in the model above to account for a difference in the outcome at baseline between the two companies.

In Sensitivity Analysis 2, we will explore post-intervention effects, using data collected at baseline, intervention, and 6 months post-intervention. In this analysis, we will assume a general time-on-treatment effect, instead of the linear effect to test different patterns of the intervention effect [39]. It is worth noting that in the Sensitivity Analysis 2 we plan to model the underlying time trend by assuming a linear effect based on existing literature and will examine this assumption using the data collected from the baseline [40,41]. These analyses will depend on model convergence and stability and aim to provide additional insights into the intervention's impact.

Given the study's small sample size, we will apply the Satterthwaite correction, which is recommended for mixed models to account for small sample size limitations, in both sensitivity analyses [42]. We will also perform model diagnostics using residual and quantile-quantile plots. All analyses will be performed using SAS (version 9.4, SAS Institute Inc, Cary, NC) and/or R (version 4.40; the R Foundation for Statistical Computing Platform).

#### 2.7.2 Power and sample size

The pragmatic single-arm hybrid effectiveness-implementation study focuses on the comparison of WHC-level study outcomes between baseline and over the 12-month period of intervention (Fig. 2). The baseline WHC-level performance for CMS165V10 is estimated at 44 % and CMS138V11 is estimated at 20 % (tobacco screening), respectively, based on previously published studies [[43], [44], [45]]. We anticipate that a 15 % improvement in these measures over the 12-month period of intervention represents a meaningful improvement, which we believe is a conservative estimate based on previous studies [46,47]. For example, one study demonstrated that similar interventions to HHM have resulted in a 28.6 % improvement in CMS165v10, which is about twice more significant than our assumption [48]. Given the nature of the study outcomes, we performed power calculations for a two-sided paired *t*-test with an alpha level of 0.05. Specifically, the improvement is expected to be 0.15 with a pooled standard deviation of 0.16, and the power calculation results revealed that we would have at least 84 % power to detect the increase in CMS165V10 from 44 % to 59 % and in CMS138V11 from 20 % to 35 % from the 12 WHCs [49].

### 2.8 Budget impact analysis and return on investment estimation

During the HHM study, the practice facilitator will document wages, benefits, and travel costs in FACITS. A spreadsheet-based cost calculator will be created based on these inputs. It will include parametric cost input models, such as staff time allocation and geographic cost adjustments, allowing users to conduct scenario analyses. These analyses will simulate the potential costs of implementing the HHM model under varying conditions, such as rural vs. urban settings, health care delivery mode, and frequency of visits. To estimate the return on investment of HHM for the employer and Medicare, we plan to apply EHR data (WHC-level) on patient demographics (age, race, gender, body mass index) and findings from the HHM study (i.e., cases of tobacco cessation, BP control before and after HHM, and patient characteristics) to the American College of Cardiology ASCVD Risk Estimator Plus to estimate potential heart diseases cases and related health care costs [49,50]. Additionally, life expectancy will be coupled with health care costs associated with heart diseases, such as hypertension and stroke, using Medicare's Diagnostic Cost Groups Hierarchical Condition Categories framework [51]. We will calculate the per-year accumulated cost by age 65 and the lifetime cost of the burden of heart disease, recognizing that the costs usually become Medicare's responsibility after an individual turns 65. To convert lifetime disease-related costs into current dollars, social discounting of 3–5% based on U.S. 30-year Treasury Bill rates will be applied. The main evaluation measure for determining return on investment is the net economic savings, calculated as the difference between the cost savings from reduced healthcare expenditures due to the HHM intervention (e.g., fewer ASCVD-related hospitalizations or reduced treatment costs) and the total input costs of conducting the intervention.

## 3 Results

Company A provided a list of 12 WHCs that met the inclusion criteria. However, as the HHM interventions specifically focus on hypertension management and tobacco cessation, three WHCs were excluded due to low smoking and hypertension rates and a small number of annual visits. This left nine WHCs eligible for randomization. Company A permitted the study team to implement HHM in seven WHCs; therefore, the remaining two WHCs act as alternative sites in case of any WHC closures during the study period. Similarly, Company B provided nine WHCs, but three were excluded due to low rates, leaving six WHCs eligible for randomization. Company B has permitted the study team to implement HHM in five WHCs; the remaining site serves as the alternative site. The WHCs excluded for low rates will not participate in the study, will not receive the intervention, and will not be in any part of the analysis.

Information on the twelve WHCs randomly selected into HHM is shown in Table 3. The average annual number of adult patient visits was 10,099 and 1,734, respectively, across the included WHCs for Companies A and B. In these WHCs, adult patient smoking rates ranged from 21 % to 59 % for Company A, and from 13 % to 24 % for Company B. The percentage of adult patients with a blood pressure of 140/90 or greater (stage 2 hypertension) ranged from 14 % to 56 % across Company A's WHCs, and from 7 % to 29 % across Company B's clinics. Among the 12 selected WHCs, two were in a rural area, and half had a town population of less than 10,000. According to data from the U.S. Census Bureau, the median average household income of the towns where WHCs were located was $48,541. The demographics of town residents were diverse across all participating WHCs, with the percentage of White residents ranging from 15 % to 89 % and the percentage of African American residents ranging from 1 % to 80 %.

**Table 3:** Characteristics of the 12 randomly selected healthy hearts in manufacturing worksite health centers.

Company	Location	Number of Adult Patient Visits^a^	Percent of Patients who are Smokers (%)	County-level Smoking Rate (%) [52]	Percent of Patients with Stage 2 Hypertension (%)^b^	County-level High Blood Pressure Rate (%)^c^ [53]	Town Population [54]	Average Town Household Income($) [54]	% of White residents(%) [54]	% of African American residents(%) [54]
Company A	WHC 1	8910	54	20	38	37	519	41,304	30	27
	WHC 2	15,994	24	24	14	36	3531	48,991	84	1
	WHC 3	7818	21	26	21	43	6384	34,086	40	50
	WHC 4	11,358	59	23	48	42	426	41,944	15	80
	WHC 5	8420	44	18	39	31	11,190	53,472	56	23
	WHC 6	5464	NA	22	26	36	2746	38,869	80	1
	WHC 7	12,732	35	22	56	38	9243	38,750	54	35
Company B	WHC 8	2447	13	8	9	27	34,470	69,889	89	3
	WHC 9	2244	24	23	29	34	2291	53,526	87	5
	WHC 10	1812	19	19	23	34	618,639	48,090	25	62
	WHC 11	1160	17	14	7	25	488,664	79,026	70	6
	WHC 12	1010	16	14	7	28	189,834	72,022	60	7

a WHCs Data collected in 2022.

b Blood pressure above 140/90 (stage 2 hypertension).

c Age-adjusted prevalence of respondents aged ≥18 years who report ever having been told by a doctor, nurse, or other health professional that they have high blood pressure.

## 4 Discussion

In this manuscript, we described the goals and design for the HHM study, an almost five-year, exploratory research program to implement and evaluate evidence-based QI interventions for hypertension and tobacco cessation in WHCs located in manufacturing sites. Upon study completion, analyses and results will reference this design paper.

WHCs, which are sponsored by employers, are well positioned to support QI and improved health outcomes for their manufacturing workers. The National Academy of Medicine and the Surgeon General have identified employers as important and underutilized partners in addressing health deficiencies [55,56]. Employers have an incentive to keep workers healthy, as healthier workers are less costly in terms of health spending [57,58]. The vast majority of large employers, including those in the manufacturing industry, offer health insurance and wellness benefits to their employees [14]. Improving the heart health of manufacturing workers and their communities would benefit the employers and would generate downstream savings for Medicare since most workers will eventually enroll in Medicare [59,60].

Further, WHCs in manufacturing communities may be an ideal health care setting for practice facilitation. WHCs tend to be small, often staffed by a nurse practitioner and medical assistant, and may not have the capacity or resources to lead QI efforts themselves [[61], [62], [63]]. Over the past decade, patient-centered outcomes research has produced new evidence-based approaches to improve the effectiveness of healthcare delivery in areas in which manufacturing communities struggle (e.g., tobacco use, cardiovascular health) [64]. However, the ability to integrate and implement these new approaches can be challenging, particularly for smaller primary care practices that do not have the staff, resources, or infrastructure for ongoing QI activities [65,66]. Although CMS has implemented a number of programs to incentivize QI in primary care clinics, including performance-based payment systems [67,68], WHCs are not eligible for those programs and do not participate in quality reporting to CMS because they primarily serve working-age populations and typically do not bill Medicare.

The study design is not without limitations. First, we were not able to collect any individual-level patient data and single monthly data (e.g., no patient IDs, confidentiality and privacy of personal data), which limited our ability to accurately account for patient population size differences across WHCs. As a result, our statistical model did not include WHC size and treated all WHCs with equal weights. We will use a size-weighted or a variance-weighted approach if the data becomes available in the future. Second, we excluded WHCs with low rates of hypertension and tobacco cessation from participating in the study, limiting the finding's applicability to these low-rate WHCs. Lastly, this study included only twelve WHCs, as it was designed as a pilot study. While the small sample size may influence the precision of the estimates and statistical power, the findings from this pilot study are expected to provide important insights and build the foundation for larger-scale studies to confirm and expand the findings. Despite these limitations, the HHM study is unique in its use of WHCs to reach manufacturing workers. It will also be a first effort to report on quality measures from WHCs. Results may reveal an economically sustainable quality improvement strategy for employers to address heart health for an at-risk population. The study will produce findings, tools, materials, and lessons that could aid efforts to implement other evidence-based quality improvement interventions in WHCs.

## CRediT authorship contribution statement

**Hanzi Jiang:** Writing – original draft, Formal analysis, Data curation. **Yao Tian:** Writing – review & editing, Validation, Methodology, Formal analysis, Conceptualization. **Jennifer Bannon:** Writing – review & editing, Conceptualization. **Amy E. Krefman:** Writing – review & editing, Conceptualization. **Lawrence C. An:** Writing – review & editing, Conceptualization. **Dustin D. French:** Writing – review & editing, Conceptualization. **Claude R. Maechling:** Writing – review & editing, Conceptualization. **Jane Holl:** Writing – review & editing, Conceptualization. **Richard Chagnon:** Writing – review & editing, Conceptualization. **Theresa L. Walunas:** Writing – review & editing, Conceptualization. **Christopher Burch:** Writing – review & editing, Data curation. **Anthony Musci:** Writing – review & editing. **Darce Latsis:** Writing – review & editing. **Dawn Carey:** Writing – review & editing. **Megan McHugh:** Writing – original draft, Supervision, Funding acquisition, Conceptualization.

## Funding statements

This work is supported by the Agency of Healthcare Research and Quality, grant number 1R18HS028782-01A1.

## Declaration of competing interest

The authors declare that they have no known competing financial interests or personal relationships that could have appeared to influence the work reported in this paper.

## Data Availability

Data will be made available on request.
