# Supplementary figures and images for: Rationale and design for Healthy Hearts in Manufacturing (HHM): A pragmatic single-arm hybrid effectiveness-implementation study for hypertension management and tobacco cessation
Source: Contemp Clin Trials Commun. 2025 Feb 3;44:101444. doi: 10.1016/j.conctc.2025.101444 (PMC11850740; doi:10.1016/j.conctc.2025.101444)

Supplementary Figure: sensitivity analysis model illustration


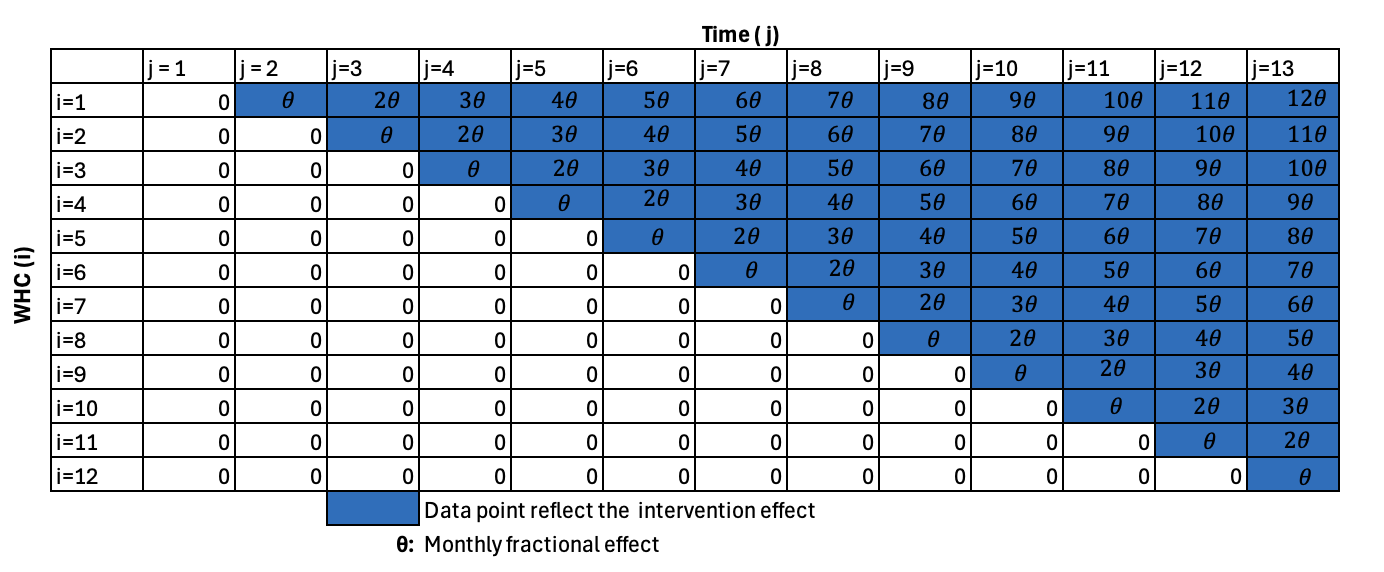


Project month 29

Project month 17

Supplement: Multimedia component 1 [file mmc1.docx]
